# Oxidation and Antioxidation of Natural Products in the Model Organism *Caenorhabditis elegans*

**DOI:** 10.3390/antiox11040705

**Published:** 2022-04-02

**Authors:** An Zhu, Fuli Zheng, Wenjing Zhang, Ludi Li, Yingzi Li, Hong Hu, Yajiao Wu, Wenqiang Bao, Guojun Li, Qi Wang, Huangyuan Li

**Affiliations:** 1 Key Laboratory of Ministry of Education for Gastrointestinal Cancer, School of Basic Medical Sciences, Fujian Medical University, Fuzhou 350108, China; wuyajiao@fjmu.edu.cn (Y.W.); baowenqiang@fjmu.edu.cn (W.B.); 2 Department of Preventive Medicine, School of Public Health, Fujian Medical University, Fuzhou 350108, China; f.zheng@fjmu.edu.cn (F.Z.); huhong1009@fjmu.edu.cn (H.H.); 3 Beijing Key Laboratory of Diagnostic and Traceability Technologies for Food Poisoning, Beijing Center for Disease Prevention and Control, Beijing 100013, China; zzwwjing@163.com; 4 Department of Toxicology, School of Public Health, Peking University, Beijing 100191, China; liludi@pku.edu.cn (L.L.); liyingzi@bjmu.edu.cn (Y.L.); 5 Department of Pathogen Biology, School of Basic Medical Sciences, Fujian Medical University, Fuzhou 350108, China; 6 School of Public Health, Capital Medical University, Beijing 100069, China; 7 Key Laboratory of State Administration of Traditional Chinese Medicine for Compatibility Toxicology, Peking University, Beijing 100191, China; 8 Beijing Key Laboratory of Toxicological Research and Risk Assessment for Food Safety, Peking University, Beijing 100191, China; 9 The Key Laboratory of Environment and Health, School of Public Health, Fujian Medical University, Fuzhou 350108, China; 10 Fujian Provincial Key Laboratory of Environment Factors and Cancer, School of Public Health, Fujian Medical University, Fuzhou 350108, China

**Keywords:** natural products, *Caenorhabditis elegans*, antioxidation, oxidative stress, reactive oxygen species

## Abstract

Natural products are small molecules naturally produced by multiple sources such as plants, animals, fungi, bacteria and archaea. They exert both beneficial and detrimental effects by modulating biological targets and pathways involved in oxidative stress and antioxidant response. Natural products’ oxidative or antioxidative properties are usually investigated in preclinical experimental models, including virtual computing simulations, cell and tissue cultures, rodent and nonhuman primate animal models, and human studies. Due to the renewal of the concept of experimental animals, especially the popularization of alternative 3R methods for reduction, replacement and refinement, many assessment experiments have been carried out in new alternative models. The model organism *Caenorhabditis elegans* has been used for medical research since Sydney Brenner revealed its genetics in 1974 and has been introduced into pharmacology and toxicology in the past two decades. The data from *C. elegans* have been satisfactorily correlated with traditional experimental models. In this review, we summarize the advantages of *C. elegans* in assessing oxidative and antioxidative properties of natural products and introduce methods to construct an oxidative damage model in *C. elegans*. The biomarkers and signaling pathways involved in the oxidative stress of *C. elegans* are summarized, as well as the oxidation and antioxidation in target organs of the muscle, nervous, digestive and reproductive systems. This review provides an overview of the oxidative and antioxidative properties of natural products based on the model organism *C. elegans*.

## 1. Introduction

### 1.1. Oxidation and Antioxidation under Physiological and Pathological Conditions

Redox homeostasis is central to life, ranging from bioenergetics to metabolism and biological functions [1]. To defend against oxidative damage, organisms have evolved defenses that primarily rely on antioxidant enzymes, the supply of their substrates and repairing the damage. Antioxidant defenses can be enhanced through physiological signaling, dietary components and potential pharmaceutical interventions, thereby improving the capacity to scavenge oxidants and electrophiles. In 1954, Commoner et al. first described the occurrence of oxidative damage in a biological environment [2]. In 1985, the concept of “oxidative stress” was first applied to the biological logic system, which is defined as any oxidative damage in which excessive production of ROS or inadequate antioxidant defense occurs [3].

An imbalance between the production of oxidants and antioxidant defenses leads to damage to biological systems. This involves the chemistry of reactions of reactive species derived from oxygen, so-called ‘oxidative stress.’ Oxidative stress has been shown to participate in a variety of diseases, including cardiovascular disease, degenerative disease and cancer, and multiple mechanisms by which oxidants contribute to cellular damage have been revealed [4,5,6]. However, the degree of oxidative stress involved in the pathology of diseases is quite variable. This variability makes it difficult to improve the antioxidant effects of therapy. Most of the antioxidant defense in cells is provided not by either exogenous or endogenous small molecules acting as scavengers but by antioxidant enzymes using their specific substrates to reduce oxidants. The therapeutic use of small molecules has been disappointing, largely due to overoptimism and incorrect assumptions about how antioxidants work [7]. Furthermore, antioxidant enzymes react with oxidants thousands to millions of times faster than small molecules and provide the predominant antioxidant defense [7,8]. Therefore, the major therapeutic opportunities lie in preventing the production of oxidants that directly damage macromolecules, inhibiting the downstream signaling of oxidants that leads to inflammation or cell death, and increasing both antioxidant enzymes and their substrates. Currently, clinical trials based on this approach are underway [4]. A greater understanding of the mechanisms of action of antioxidants and where and when they are effective may provide a rational approach to addressing oxidative stress.

### 1.2. Role of Oxidation and Antioxidation Based on the Complex Composition of Natural Products

In the pharmaceutical industry, the approved medicinal products are mostly composed of a single molecule or combinations of single molecules whose pharmacological properties and safety are characterized during the preclinical phase and then validated in human trials [9]. The plants, animals, fungi, bacteria and archaea are a source of drugs of natural origin. Each of them contains hundreds of compounds, which belong to different classifications, such as diterpenoid, flavonoid, coumarin, steroid, hydrocarbon, carboxylic acid, ester, aldehyde, alcohol, ketone, ether, epoxide, phenol and so on [10]. Although many approved drugs isolated from natural sources have been proven to be involved in oxidation or antioxidation in experimental models and human studies [11], their mechanisms of action are usually not yet fully elucidated since it is difficult to identify the effective compounds and dosages. For example, researchers have demonstrated that *Glycyrrhizae radix* extract is beneficial in preventing oxidative damage in *Caenorhabditis elegans* (*C. elegans*). However, this extract is a mixture of all water-soluble compounds from *Glycyrrhizae radix*, so it is still unknown which compound plays an antioxidant role in this protective effect [12]. The composition of natural products is complex, thus posing a challenge to clarify their role in the oxidation and antioxidation process clearly. Along with the improved sensitivity of trace detection techniques (e.g., high-resolution mass spectrometry, instrumental neutron activation analysis, atomic absorption spectroscopy, ultra-performance liquid chromatography), an increasing number of newly discovered compounds have been isolated from natural products [13]. By means of plane and spatial structure analysis methods (e.g., nuclear magnetic resonance, atomic spectroscopy, circular dichroism spectrum, single crystal X-ray diffraction), the structures of these novel compounds can be resolved, making it possible to reveal the oxidation and antioxidation effects and mechanisms [14,15].

### 1.3. Advantages of C. elegans as a Model of Oxidation and Antioxidation Assessment

*C. elegans* was introduced to science as a model organism for development and neurobiology in 1965 [16]. Nematodes share approximately 60–80% of genes and 12 signaling pathways with humans [17,18], and notes on gene function can be obtained from the WormBase online consortium. *C. elegans* shows many advantages, such as self-fertilization, a short life cycle, a small and transparent body, ease of culture, simple operation and low cost, without limitations in ethics, showing great potential as an alternative model for the 3R principle [19,20]. Despite their simple structure, nematodes have complete muscle, subcutaneous tissue, nervous system, gut, gonads, glands and excretory system, and many basic physiological processes and oxidative stress responses of higher organisms are also highly conserved in nematodes [21,22]. Therefore, nematodes have great potential as models for evaluating the pharmacological and toxicological effects in humans [23], and we systematically summarize the advantages and disadvantages of *C. elegans* in the pharmacotoxicology research field and compare this model with other classical models, namely *Drosophila*, zebrafish, yeast, cell and mammalian in Table 1. In recent decades, nematodes have become very popular for high-throughput drug screening. This allows the drug discovery process to be studied throughout the life cycle and the manipulation of individual genes or genomes, such as *N*-ethyl-*N*-nitrosourea or ethyl methanesulfonate mutations, RNA interference (RNAi) and CRISPR [24]. Nematodes have been applied to the mechanism of action of addictive drugs [25], the pharmacological effects of neurodegenerative diseases such as Alzheimer’s disease (AD) drugs [26], and the neurotoxicity of anticancer drugs [27]. Currently, a large number of mutants have been used to study the molecular mechanism of effective components of natural products. For example, ursolic acid affects the stress response of nematodes by disturbing genes expressions of dopamine receptors [28,29], as also applies in the toxicological mechanism of the extract of *Peganum harmala* L. seeds [30], and the intestinal toxicity mechanism of *Euphorbia* factor L1 [31], etc. In addition, there are many reports on the effectiveness of antioxidants in *C. elegans*, as they can be used to establish an antioxidant stress response model for the assessment of antioxidant capacities in vivo [32,33,34,35].

An oxidant is formed as a normal product at a relatively low concentration during aerobic metabolism but is produced at ascending rates under pathological conditions. A quasi-steady state is maintained via intricate patterns of oxidant and antioxidant balance, and the disruption will jeopardize normal biological functions [36,37]. Natural products represent rich sources of bioactive compounds for pharmacology and toxicology, and phytochemicals show great potential in oxidation or antioxidation, as proven by numerous experimental models and human studies. Moreover, aqueous and organic solvent extracts, as well as secondary metabolites, play vital roles in oxidative stress by means of different cellular and molecular mechanisms. As a rapidly developed model organism, *C. elegans* has been introduced into the oxidation and antioxidation assessment of natural products for decades. The results based on *C. elegans* correlate with traditional experiments on rodents and rabbits [38]. In this brief overview, we will mainly discuss the oxidation and antioxidation induced by natural products and focus on the model of *C. elegans* to provide insights into the oxidative stress theory.

## 2. Establishment of Oxidative Stress Model in *C. elegans*

The lifespan of *C. elegans* is always proportional to its resistance to environmental stress, and stress resistance is dominant in the lifespan of *C. elegans* [39]. Under heat stress, cells show a heat shock response and induce gene expression to prevent cell degeneration and enhance heat resistance. Heat shock experiments usually observe the survival time of nematodes by placing them at a temperature higher than the suitable living environment for some time. This index has been widely used to evaluate the protective effect of substances. For example, Lin et al. exposed nematodes to traditional Chinese herbal tea for 4 days, transferred them to 35 °C and recorded death per hour, indicating this treatment extended the average lifespan of nematodes significantly [40]. The same method was also used to evaluate the antistress effects of *Cyclocarya paliurus* polysaccharide leaf extract and piceatannol, and the authors came to similar conclusions [41,42]. There are also some different treatment methods in heat stress experiments; for example, Lu et al. studied the protective effect of calycosin on nematodes under heat stress. On the third day, adult nematodes were cultured at 36 °C for 4 h and then transferred to 20 °C, and their survival was recorded every day [43].

The oxidative stress model of nematodes involves observing the survival of nematodes by exposing them to strong oxidants. Paraquat is a common stimulator of oxidative stress. Nematodes were exposed to NGM plates containing 5–50 mM paraquat, and the survival rate was monitored every 12 h [40,41,42,44]. Alternatively, the paraquat concentration was adjusted to 300 mM, and the survival time was recorded every 1 h [45]. The hydrogen peroxide (H_2_O_2_) test can also be used for the oxidative stress model. Nematodes were exposed to 0.8 mM H_2_O_2_ for 7 h to calculate the survival rate or incubated in 4–12 mM H_2_O_2_ for 4 h, and the number of surviving nematodes was recorded every 30 min [40,41,46,47]. Oxidative stress can also be tested using juglone, exposing nematodes to lethal juglone at 250 μM for 3 h, 300 μM for 1 h or 150 mM for 24 h, and then the survival rates of nematodes were recorded [28,29,48,49].

The heat shock response in *C. elegans* has revealed three related neuroendocrine signaling pathways: the nuclear hormone receptor pathway, transforming growth factor-β pathway and IGF/insulin-like signaling pathway [50]. Among them, the IIS pathway is the most thoroughly studied and has been demonstrated to play an important role in the signaling regulation of oxidative stress in *C. elegans*. Lin et al. and Shen et al. proved that the improvement of stress resistance mediated by phytomedicine was positively correlated with the activation of IIS pathway [41,42]. To investigate whether forkhead box protein O (DAF-16), a key regulator of antioxidation or heat stress, plays a role in this process, the subcellular distribution of DAF-16 in the TJ356 mutant was observed. The transfer of DAF-16 from the cytoplasm to the nucleus could be inhibited under stress [40]. The mechanism of *Cyclocarya paliurus* (*C. paliurus*) polysaccharide enhancing nematode heat tolerance was related to heat shock transcription factor 1 (HSF-1) without affecting the expression of DAF-16 in TJ356 but changed the fluorescence expression of SOD-3::GFP, and altered the expression of heat stress-related genes *hsp-16.1* and *hsp-16.2*, suggesting that the HSF-1 pathway was necessary to improve heat tolerance. The longevity promoter skinhead-1 (SKN-1) regulates oxidative stress resistance. Under H_2_O_2_-induced oxidative stress, *C. paliurus* polysaccharide did not shorten the longevity of SKN-1 mutants, suggesting that the *C. paliurus* polysaccharide-mediated oxidative stress is dependent on SKN-1 [41].

## 3. Biomarkers of Oxidation and Antioxidation of Natural Products in *C. elegans*

### 3.1. Reactive Oxygen Species

**Figure 1:**
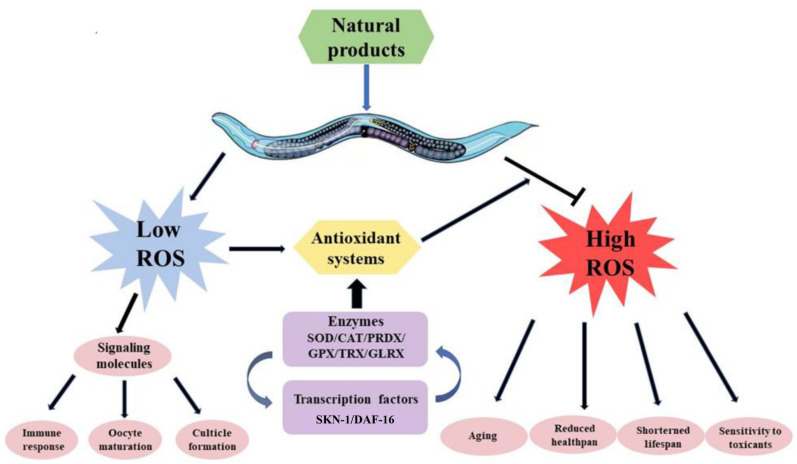
ROS-induced oxidative stress in *C. elegans*. Natural products could induce ROS in worms. At low concentrations, ROS act as signaling molecules to regulate the immune response, oocyte maturation and cuticle formation. When ROS reach a certain threshold, they result in aging, reduced healthspan, shortened lifespan and increased sensitivity to toxicants. On the other hand, antioxidant systems are activated by ROS, such as related enzymes (SOD, CAT, etc.) and transcription factors (SKN-1/DAF-16) to fight against ROS-induced toxicity.

ROS, such as H_2_O_2_, hydroxyl radical (HO^•^) and superoxide anions, are highly reactive molecules constantly generated during aerobic metabolism. The main site of ROS production is the mitochondria, especially the electron transport chain. Furthermore, ROS formation can be induced by endogenous and exogenous compounds or stressors [51]. As shown in Figure 1, at low concentrations, ROS play physiological roles in various cellular processes as signaling molecules. In nematodes, ROS production triggered by major sperm protein mediates oocyte maturation [52]. Moreover, the final step of collagen maturation to form the cuticle involves ROS participation [53] and thus is essential for efficient molting and larval survival [54]. Moreover, ROS can be induced by pathogen infection to kill invading bacteria and fungi [55,56]. Nevertheless, excessive ROS result in a shortened lifespan, reduced healthspan and sensitivity to toxicants [57]. Aging is proposed to be a consequence of the progressive accumulation of oxidative damage caused by ROS generated during the lifetime of an organism [58]. Nevertheless, diverse studies on genes encoding ROS-detoxifying enzymes have failed to demonstrate a direct link between ROS and aging (reviewed in [59]). Considering that the natural habitat of *C. elegans* contains a much lower level of oxygen than ambient laboratory conditions [60], it is suggested that only when the ROS level reaches a certain threshold can it become detrimental and shorten the lifespan [61].

There are well-established probes and methods to evaluate ROS levels in *C. elegans*. The 2′,7′-dichlorofluorescein diacetate (H_2_DCF-DA) probe has been extensively used to examine endogenous ROS levels, where nonfluorescent H_2_DCF is converted to highly fluorescent DCF at 485/535 nm in the presence of ROS [62]. This reaction is sensitive to H_2_O_2_, HO^•^ and ROO^•^, but not to ^•^NO, HOCl or O_2_^•−^ [63]. Mitochondrial ROS can be detected by MitoTracker Red CM-H_2_XRos or MitoSOX, which are more specific to H_2_O_2_ and O_2_^•−^, respectively [64,65]. The H_2_O_2_ redox sensor HyPer can be used throughout the lifespan of worms to monitor protein oxidation states and peroxide levels [66]. The employment of reporter strains or RNA silencing approaches could uncover the effect of environmental chemicals and natural products.

### 3.2. Antioxidant Enzyme and Transcription Factors

When worms encounter oxidative stress, ROS-detoxifying enzymes are recruited to fight against the situation. Antioxidant enzymes, such as superoxide dismutase (SOD), catalase (CAT), peroxiredoxins (PRDX), glutathione peroxidases (GPX), thioredoxins (TRX) and glutaredoxin (GLRX) are expressed and highly conserved in *C. elegans*. Superoxide is removed by SOD, resulting in the production of H_2_O_2_, which can be further detoxified to water by PRDX, CAT and GPX. Peroxidase activity is recovered by TRX or GLRX [67]. Enzyme activities can be detected by chemiluminescent or absorbance assays [68]. Moreover, the mRNA levels of the enzymes can be easily monitored by qRT-PCR or transgenic reporter strains [69,70].

Natural components isolated from *Heterotheca inuloides* have shown the ability to inhibit lipid peroxidation, scavenge oxidants and nonbiological free radicals, and possess anti-inflammatory activity via the activation of antioxidant enzymes, resulting in the elongation of the nematode life span [71,72]. The extract of *Lonicera japonica* positively regulated SOD-3 to promote healthy aging [73]. Alongside the increase in SOD activity, the increased CAT activity induced by orange extracts is correlated with a reduction in malondialdehyde (MDA) level [74]. In addition, polysaccharides from *Bergenia emeiensis* reduced the ROS level significantly, codominant with the overexpression of sod-3 and the increased nuclear localization of the daf-16 transcription factor [75].

As mentioned above, transcription factors (such as SKN-1 and DAF-16) mediate the induction of antioxidant and stress-protective enzymes to fight against ROS [76,77]. The effects of natural products can be investigated by taking advantage of *C. elegans*; for example, transgenic strains such as DAF-16::GFP and SKN-1::GFP can be utilized to check the localization of transcription factors, and the involvement of these factors can be further studied using loss-of-function strains and RNAi. Flavonoids and their extracts, such as quercetin, myricetin and nobiletin, exert life-prolonging effects through the translocation of DAF-16 [32,46,78]. *Hibiscus sabdariffa* L. extract positively affects lifespan depending on the activation and nuclear localization of DAF-16 and SKN-1 [79].

### 3.3. Glutathione Levels

Glutathione (GSH) is the most important cellular redox buffer, consisting of a reduced form of GSH and an oxidized form of GSSG. Under normal conditions, most GSH molecules present as reduced GSH, while disulfide GSSG remains minimal. GSH is normally referred to as the major antioxidant of the cell and other functions such as detoxification, modulation of cell proliferation, storage and transport of cysteine [80]. The ratio of GSH:GSSG is a key indicator of the redox environment, owing to the GSH pool being three to four orders of magnitude higher than other redox couples [81,82].

GSH in worms can be detected by spectrophotometric or high-performance liquid chromatography (HPLC) analysis after reaction with 5,5′-dithiobis-(2-nitrobenzoic acid) (DTNB) or ortho-phtaldialdehyde (OPA) [45,83]. If the total GSH (i.e., GSH + GSSG) needs to be determined, GSH reductase can be used with NADPH to recycle GSSG into GSH prior to detection [84]. In addition, Grx1-roGFP1 and Grx1-roGFP2 fluorescent probes can determine the GSSG/2GSH ratio in worms [85,86]. *Momordica* saponin extract improves the healthspan of nematodes through its antioxidant activities, including the elevation of GSH/GSSG induced by H_2_O_2_, paraquat and heat, mediated by *skn-1* and *hsf-1* [87]. Similarly, polysaccharide extracted from the leaves of *C. paliurus* enhanced stress resistance in worms, as demonstrated by reduced peroxidation products (ROS, MDA, GSSG) and the upregulation of SOD, CAT, GSH via *skn-1* and *hsf-1* [41].

### 3.4. Oxidative DNA Damage and Repair

Under normal physiological conditions of aerobic organisms, a sophisticated and elaborate balance mechanism is maintained between endogenous oxidants and enzymatic or nonenzymatic antioxidant defenses. Otherwise, excessive ROS accumulation will lead to oxidative damage to biomolecules, including DNA, lipids and proteins, of which oxidative DNA damage contributes to the pathology of genetic instability and accounts for genetic diversity [88]. The types of oxidative DNA damage include base modifications, single strand breaks (SSB), double strand breaks (DSB), and non-DSB oxidatively generated clustered DNA lesions (OCDL), all of which are associated with the pro-death signaling pathway. Thus, DNA damage and repair have been the primary foci of interest in natural product-induced oxidative stress [89].

HO^•^ is the most important oxygen-free radical that causes oxidative DNA damage. It is mainly produced by the Fenton reaction of H_2_O_2_, metals, and endogenous or exogenous ROS. Once HO^•^ is produced near DNA, it will attack the nucleobases of DNA strands to cause the formation of C8-hydroxyguanine (8-OHGua) or its nucleoside from 8-hydroxy-2′-deoxyguanosine (8-OHdG). Then, 8-OHdG undergoes keto-enol tautomerism, thereby generating the oxidized product 8-oxo-7,8-dihydro-2′-deoxyguanosine (8-oxodG) [90]. 8-OHdG is the predominant biomarker of nuclear and mitochondrial DNA damage. Differing from protein and lipid oxidation, oxidative damage DNA repair requires nucleotide replacement, whereby 8-OHdG is excreted by cells. The most widely used methods to quantitatively detect the 8-OHdG level in *C. elegans*, including HPLC, tandem mass spectrometry (MS-MS), gas chromatography–mass spectrometry (GC–MS), electrochemical detection (EC) and enzyme-linked immunosorbent assay (ELISA), are suggested [91].

*C. elegans* is a commendable model for studying DNA damage or repair induced by natural products [92]. Aflatoxin B1 (AFB1), the most toxic mycotoxin produced by *Aspergillus flavus* and *Aspergillus parasiticus*, is mainly metabolized by CYP450 into the genotoxic metabolite 8,9-epoxide-AFB1. For AFB1-induced genotoxicity research, a study reported that AFB1 inhibited the growth and reproduction of *C. elegans* and triggered a high DNA lesion frequency per 10 kb. DNA damage can be repaired by the nucleotide excision repair (NER) system, and AFB1-induced development and DNA toxicity were more obvious in the NER-deficient *xpa-1* gene knockout strain RB864 than in the wild-type N2 strain. The *cep-1*, *hus-1*, *egl-1* and *clk-2* pathways for the DNA damage checkpoint were activated and associated with AFB1-induced apoptosis [93]. Regarding the protective effect of natural products on DNA damage, both quercetin and *Rosa rugosa* aqueous polyphenol reduced Fenton’s reagent-induced pUC18 plasmid DNA degradation from the supercoiled form into a linear or open circular form [94,95].

### 3.5. Lipid Peroxidation

Lipid is an essential component of cellular and organelle membranes to maintain morphological structure and biological processes [96]. As the primary target of free radical species attack, membrane lipid bilayer peroxidation is associated with a variety of pathological states, such as diabetes, cardiovascular diseases of hypertension, atherogenesis and stroke, acute and chronic kidney injury, and neurodegenerative AD and Parkinson’s disease (PD) [97,98]. Polyunsaturated fatty acids (PUFAs), long-chain fatty acids with more than one double bond, including linoleic acid (C18:2), α-linolenic acid (C18:3) and arachidonic acid (C20:4), are distributed throughout the body. The PUFAs most susceptible to peroxidation have the structural characteristic of containing a (1Z, 4Z) pentadiene moiety [99]. Lipid peroxidation reactions are free radical species such as HO^•^, peroxyl radicals (LOO^•^) that remove electrons from lipids to produce reactive intermediates for further reactions. This process is classified into three steps: initiation, propagation, and termination, and is first initiated by chemical reactions or enzymatic catalysis (lipoxygenases, cyclooxygenases, cytochrome P450). Prooxidants such as HO^•^ abstract the allylic hydrogen atom, an atom of the methylene group located between two olefinic bonds of PUFAs, to form the carbon-central lipid radical. After molecular rearrangement, the carbon radicals are stabilized gradually to form a conjugated diene. In the propagation phase, one molecule of diradical O_2_ attacks the weak C-H bond and disrupts its stability to form LOO^•^. In the termination phase, antioxidants provide a hydrogen atom to LOO^•^ in favor of the formation of nonradical products, such as MDA, 4-hydroxy-nonenal (HNE), F_2_-isoprostane 15(S)-8-*iso*-prostaglandin F_2α_ (15(*S*)-8-*iso*-PGF_2α_) and other F_2_-isoprostanes [100]. Interestingly, lipofuscin, a heterogeneous composition consisting of oxidized proteins and lipids, is a specific biomarker to assess lipid peroxidation in *C. elegans* [101].

The protective and adverse effects of various compounds or extracts from natural products on lipid peroxidation were evaluated based on the *C. elegans* model. For example, Nile red staining and the determination of triglycerides showed that the ethyl acetate extract of *Punica granatum* fruit peel (PGPE) inhibited lipid accumulation and extended the life span of L4 nematodes. The hypoglycemic activity was similar to that of acarbose. GC-MS analysis of PGPE detected 48 compounds, of which 5-hydroxymethylfurfural and 4-fluorobenzyl alcohol were the most biologically active components, with a maximum area value of 48.59% [102]. In another study, ferulsinaic acid, a newly rearranged class of sesquiterpene coumarin from the genus *Ferula*, prolonged the mean life span of *C. elegans* N2 from 18.64 days to more than 20 days and alleviated paraquat-induced oxidative stress. Meanwhile, the underlying mechanisms involve ferulsinaic acid attenuating both the lipid peroxidation product MDA and the formation of the advanced glycation end product *N^ε^*-carboxymethyllysine [103]. Kim et al. reported neutral and acidic polysaccharides from *Panax ginseng* C.A.Mey., a perennial herb of the family Araliaceae, exhibited excellent reducing power, ferrous ion chelating capacity, scavenging activity of 2,2′-azino-bis-(3-ethylbenzothiazoline-6-sulfonic acid, hydroxyl peroxide and ROS, and ferrous chelating, and significantly inhibited MDA production [104].

### 3.6. Protein Oxidation

Proteins have an overwhelming dry mass percentage of 70% in cells and tissues. When oxidation occurs, the reaction rate depends on the concentration of the biological targets, multiplied by the rate constant. Proteins have a high risk of oxidative damage since they are present in high concentrations of up to 1–3 mM in plasma and 5–10 mM in cells and have a high rate constant for oxidative reactions [105]. Of course, this is a simplified process to calculate the protein oxidation rate without consideration of the biofilm barrier, microenvironments, molecular transfer reactions and secondary reactions.

The origin species for protein side chain oxidation are varied, including radical oxidants of alkyl radical (R^•^), alkoxyl radical (RO^•^), peroxyl radical (ROO^•^), thiyl radical (RS^•^), sulfinyl radical (RSO^•^), thioperoxyl radical (RSOO^•^), disulfide anion radical (RSSR^•−^), superoxide radical anion ($O_{2}^{}$), HO^•^, hydroperoxyl radical (${\mathrm{HO}}_{2}^{}$), carbonate radical anion (${\mathrm{CO}}_{3}^{}$), nitric oxide (NO^•^), and nonradical two-electron oxidants such as hypochlorous acid (HOCl), H_2_O_2_, peroxynitrous acid (ONOOH), singlet oxygen (^1^O_2_), O_3_, ${\mathrm{NO}}_{2}^{+}$, N_2_O_2_, HNE, deoxyosones, ketoamines, quinones and aldehydes [106]. The main amino acid targets are Cys, Met, Trp, Tyr, His, Sec, Arg, α-amino, cysteine, and so forth, among which Cys, His and Sec are pH-dependent, presenting high reactivity with ionized radical species. Radicals, nonradical oxidants, and metal-oxo complexes can modify proteins to induce various posttranslational modifications, thus changing the amino acid composition, protein structure, folding and assembly, charge, hydrophilia, degradation and even function [107]. The defense system of protein oxidation is divided into two types: enzymes and proteins, including SOD, CAT, GPx, glutathione S-transferase (GST), ceruloplasmin, ferritin, and transferrin, and metabolites and vitamins, such as lipoic acid, NAD(P)H/NAD(P), bilirubin, uric acid, vitamins A, C and E [108].

To detect the reactive intermediates on proteins after oxidation, nitroso spin trap, iodide, coumarin boronic acid, 5-thio-2-nitrobenzoic acid, and dimedone can be used for chemical reactions. Their products can be detected by electron paramagnetic resonance, Western blotting, ELISA, UV absorbance, and mass spectrometry. For the assessment of protein amino acid composition and modification, proteins should be isolated and digested for free amino acids or oxidation products after HPLC separation, mass spectrometry, fluorescence probe, electrochemistry and UV are applied for analysis and quantification [109].

Carbonylation is one of the representative irreversible forms of oxidative damage to proteins. This posttranslational modification is initialized by MDA, HNEand hydroxyl-hexanal (HHE). It attacks the side chains of lysine, cysteine and histidine, yielding reactive carbonyl moieties in proteins, such as aldehydes, ketones and lactams. As a result, carbonylated proteins usually lose their functions [110]. Pitanga, the fruit of the pitangueira tree (*Eugenia uniflora* L.), exhibited protective effects against H_2_O_2_-mediated ROS accumulation and reduced the content of protein carbonyl induced by oxidative damage [111].

### 3.7. Fluorescent Labeling of Oxidation Related Proteins

When a transgenic animal model is constructed, a reporter gene of β-galactosidase (LacZ) or green fluorescent protein (GFP) is usually used to judge whether the target genetic modification is successful. As a model organism with transparent and thin bodies, fluorescent labeling is the perfect method to fuse target proteins to directly visualize protein expression [112]. To date, hundreds of fluorescently labeled nematodes have been obtained from biological resource sharing platforms, including GFP, enhanced GFP (eGFP), yellow fluorescent protein (YFP), red fluorescent protein (RFP), and blue fluorescent protein (BFP) labeling.

To study the oxidative effects and molecular mechanisms in *C. elegans*, multiple antioxidant enzymes were labeled with GFP. For example, to detect H_2_O_2_ production in *C. elegans*, strain JV1 with the H_2_O_2_-specific biosensor Hyper was constructed by means of the fusion between circularly permuted yellow fluorescent protein (cpYFP) and the regulatory domain of the prokaryotic *Escherichia* *coli* H_2_O_2_-sensitive transcription factor OxyR [113]. For the assessment of the oxidative stress state, GFP-labeled DAF-16 strain TJ356 was used to observe the localization of DAF-16 according to its subcellular distribution in nuclear, cytosolic and intermediate [114]. Previous research reported that the leaf extract of *Caesalpinia mimosoides* enhanced oxidative stress resistance via the DAF-16 pathway by improving DAF-16 translocation into the nucleus [115]. To evaluate the antioxidant system, the transgenic strains CF1553, CF1580, CF1588, CF1660, CF1874, MAH99 and SD1746 were all sod-3p::GFP phenotypes with different genotypes. In marine drug research, after treatment with peptides SeP2 and SeP5, separated from the hydrolysate of *Sepia esculenta*, sod-3p::GFP expression in CF1533 nematodes was significantly increased, indicating activation of the antioxidant system. Consistent with the suppression of paraquat-induced ROS and MDA elevation, sod-3 gene expression was upregulated in wild-type N2 worms [115]. Another antioxidant enzyme, GST, plays a vital role in detoxifying oxidative damage, has been labeled with GFP in strains CL2166, CL6180, FT1459, OH2204, QV224, SPC167 and VP596, and is widely used in antioxidant effect studies of natural products [116,117]. Furthermore, the transcription factor protein SKN-1, the orthologue of human nuclear factor erythroid 2-related factor 2 (Nrf2), regulates the expression of phase II detoxification enzymes to exert antioxidation and xenobiotic defense capacity and has corresponding GFP-labeled strains LG326, LG340, LG348, LG357 and SYS81 [118].

## 4. Signaling Pathways Involved in the Oxidation and Antioxidation of Natural Products in *C. elegans*

**Figure 2:**
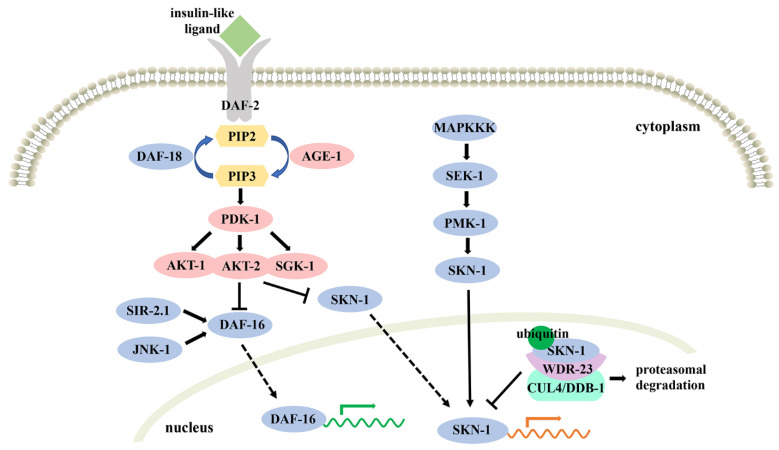
Schematic diagram of the IIS and SKN-1/Nrf2 pathways in *C. elegans*.

There is solid evidence that molecular mechanisms involving oxidative stress are considerably conserved in evolution. For example, the insulin/insulin-like growth factor-1 (IGF-1) signaling (IIS) pathway and Nrf2 system are structurally and functionally conserved in *C. elegans*, *D. melanogaster*, and mammals [119,120,121,122]. Due to the advantages of *C. elegans*, it has become a promising and powerful model to explore the mechanism of action underlying oxidative stress and evaluate the antioxidant effects of natural products [19,123]. Here, we review the two most important pathways, the IIS and SKN-1/Nrf2 pathways, related to the oxidative stress in *C. elegans* (Figure 2). Additionally, we illustrate several natural products possessing antioxidative effects via these two pathways, which provide clues for the therapy of human diseases.

### 4.1. IIS Pathway

The IIS pathway plays a vital role in various biological processes in *C. elegans*, including fat metabolism, reproductive maturation, longevity, diapause and stress resistance [124]. Researchers noticed that *daf-2* (homologous gene of the human insulin/IGF-1 receptor) and *age-1* (homologous gene to the human phosphatidylinositol-3-OH kinase, PI3K) mutants not only possess the characteristics of longevity and dauer formation but also exhibit high resistance to oxidative stress through upregulation of the activities of SOD, CAT and GST [125,126,127,128,129]. In the IIS pathway, DAF-2 is activated by binding an insulin-like ligand, phosphorylates DAF-2, and recruits and activates its downstream target AGE-1 to catalyze the formation of PIP3 from PIP2, whereas DAF-18 (homologous with the human tumor suppressor PTEN) antagonizes the process. Subsequently, PIP3 activates 3-phosphoinositide-dependent protein kinase 1 and in turn results in phosphorylation and activation of serine/threonine kinases AKT-1, AKT-2 and SGK-1 [130,131,132]. Consequently, the principle downstream target DAF-16 (homologous with the human forkhead transcription factor) is phosphorylated and inactivated, failing in nuclear translocation and the transcriptional regulation of plentiful effectors involved in stress resistance, lifespan, fat storage and so on [133]. Remarkably, the effects induced by the *daf-2* and *age-1* mutations are suppressed by the *daf-16* mutation, which means that the activation of the IIS pathway is dependent on the antagonism of DAF-16 [134,135]. Furthermore, two key modifiers, SIR-2.1 (homologous with the human NAD^+^-dependent histone deacetylase) and JNK-1 (homologous with the human c-Jun N-terminal kinase) take part in the regulation of downstream genes via active DAF-16 by posttranslational control [136,137].

Several natural compounds have been demonstrated to manifest antioxidant activity via the IIS pathway in *C. elegans*. Icariin is the main active flavonoid glycoside of *Herba epimedii*, which has many pharmacological effects, such as anti-osteoporosis, neuroprotection, cardiovascular and sexual function improvement [138]. It was found that icariin and its active metabolite icariside II could protect *C. elegans* against oxidative stress and prolong lifespan in wild-type N_2_ but not in *daf-2* and *daf-16* mutants. Moreover, the transcriptional level of the *daf-2* target *sod-3* was significantly increased after treatment with icariside II [139]. Caffeoylquinic acids, plant-derived polyphenol compounds, are abundant in coffee beans, tea, and herbs. Research has shown that 3,5-dicaffeoylquinic acid, a caffeoylquinic acid, can inhibit *daf-2* and *age-1*, upregulate *daf-16*, promote its nuclear localization, and increase the expression level of *sod-3* to protect against oxidative damage [140]. As another example, sappanone A, which is a homoisoflavonoid compound isolated from the traditional Chinese medicine *Caesalpinia sappan* L., was able to reduce intracellular ROS generation and lipofuscin accumulation in *C. elegans*. In addition, sappanone A treatment decreased the gene expression of *daf-2* but increased the gene expression of *daf-18* and *daf-16*, which implied that the IIS pathway might contribute to antioxidation by sappanone A [141].

### 4.2. SKN-1/Nrf2 Pathway

As one of the important transcription factors that modulate the IIS pathway in *C. elegans*, SKN-1, the orthologue of human Nrf, acts as a protective protein to counteract oxidative damage [142]. The Kelch-like ECH-associated protein 1 (Keap1)-Nrf2 system is a defense mechanism against environmental insults in mammals. Under nonstress conditions, Keap1, which is also called the sensor molecule, directly binds with Nrf2 and inhibits its activity by the ubiquitin-proteasome system. However, when attacked by electrophiles or ROS, the stability of the Keap1-Nrf2 system is impaired. Nrf2 translocates to the nucleus and subsequently binds to antioxidant responsive elements with a heterodimeric partner, small Maf, to induce a battery of target genes encoding antioxidant proteins [121,143]. In *C. elegans*, SKN-1 is expressed constitutively in the nucleus of two ASI chemosensory neurons and accumulates in the nuclei of intestinal cells just during exposure to oxidative stress [144]. Characteristically, SKN-1 uniquely binds to specific DNA sequences as a monomer due to the absence of the basic leucine zipper (ZIP) dimerization module, which is essential to interact with small Maf [145]. *C. elegans* also lacks the orthologue of Keap1. Under unstressed conditions, the nuclear translocation of SKN-1 is inhibited via phosphorylation by glycogen synthase kinase 3 and the IIS kinases AKT-1, AKT-2 and SGK-1 [146,147]. Moreover, once SKN-1 enters the nucleus of unstressed cells, the WD40 repeat protein WDR-23 recruits SKN-1 to the CUL-4/DDB-1 ubiquitin ligase and suppresses its accumulation through proteasomal degradation [148]. However, under oxidative stress conditions, the p38 mitogen-activated protein kinase (MAPK) cascade is involved and activated. Briefly, MAPKKK phosphorylates SEK-1 (MAPKK), SEK-1 phosphorylates PMK-1 (MAPK), and then PMK-1 phosphorylates SKN-1 at Ser74 and Ser340, leading to its nuclear localization. Consequently, SKN-1 in the nuclei activates stress-induced genes (e.g., *gcs-1*) encoding the phase II detoxification enzyme in response to oxidative stress [149,150].

Many bioactive natural compounds prevent oxidative damage and promote health via the SKN-1/Nrf2 pathway. For example, garlic characteristic organosulfur compounds (OSCs) are considered beneficial to multiple chronic diseases. It was shown that two main water-soluble OSCs, S-allylcysteine (SAC) and S-allylmercaptocysteine (SAMC), could selectively induce SKN-1 activity by WDR-23 regulation to reduce the accumulation of ROS and enhance oxidative stress resistance [151]. Wedelolactone (WDL), a kind of naturally occurring coumestan in *Eclipta alba*, has been found to elevate the mRNA expression levels of various stress-responsive genes and suppress the production of ROS, lipid peroxidation and protein carbonylation through SKN-1, which ultimately leads to mitigation of Parkinsonism [152]. Furthermore, velvet antler, a famous Chinese medicine, exerts therapeutic effects on many diseases, such as mammary hyperplasia and uterine fibroids [153]. It was found that velvet antler methanol extracts could reduce oxidative damage and prolong the healthspan of *C. elegans* by promoting SKN-1 nuclear translocation and upregulating the expression levels of downstream antioxidative genes, including *gst-4*, *gst-7*, *gst-10* and *sod-3* [154].

As mentioned above, the IIS and SKN-1/Nrf2 pathways play important roles in stress resistance. In addition, other signaling pathways, such as the JNK MAPK pathway and target-of-rapamycin (TOR) pathway, also influence oxidative stress through complicated crosstalk with the IIS and SKN-1/Nrf2 pathways [155]. Given that the signaling pathways are diverse and intricate, comprehensive and in-depth studies are still needed in the future.

## 5. Natural Products-Induced Oxidation and Antioxidation in the Target Organs of *C. elegans*

### 5.1. Muscle

Abnormal ROS production and redox imbalance play an essential role in age-related loss of muscle mass and function [156]. The muscle function of *C. elegans* is mainly expressed as pharyngeal pumping, an important marker for aging in *C. elegans* [157,158]. Numerous natural antioxidant products capable of lifespan extension can contribute to muscle health [159,160,161], suggesting that these compounds possess antiaging effects on muscle cells. They can perform functions through different pathways associated with oxidative stress. Tomatidine, a natural compound abundant in unripe tomatoes, can enhance pharyngeal pumping and extend the lifespan of *C. elegans*. Appropriate amounts of tomatidine can moderately increase ROS levels, which are essential to trigger mitophagy and activate adaptive cellular stress responses, thus protecting muscle cells against metabolic and oxidative stress to counteract age-related dysfunction and degeneration [162]. Similarly, Liangyi Gao, a traditional Chinese medicine that has antioxygenic activity, delays age-associated pharyngeal contraction decline by elevating antioxidant enzyme activity to eliminate oxygen free radicals via the activation of *sod-3* [163]. In addition, Chauhan et al. [164] studied the effect of the natural antioxidant *Moringa* *oleifera* extract on the muscle health of *C. elegans* by measuring pharyngeal pumping, body bending, and reversal frequency and found that this compound had the potential to improve muscle function mediated by *daf-16*. Moreover, *Glochidion zeylanicum* leaf extracts can also modulate oxidative stress via the DAF-16/FoxO and SKN-1/Nrf-2 signaling pathways, leading to lifespan extension and the enhancement of pharyngeal contraction in *C. elegans* [165].

### 5.2. Nervous System

Although worms are very different from humans, the nervous systems of the two are highly conserved, constructed by one-third of the cells in *C. elegans*. This makes worms a feasible tool to investigate natural product-induced redox responses in the nervous system, including neuroinflammation [166], PD and AD [167,168] and so on. Neurotoxicity in worms can be monitored according to behavioral endpoints such as locomotion and foraging ability, morphological and physiological endpoints such as neuronal structure, neurodegeneration and neuroinflammation, and molecular endpoints such as acetylcholinesterase activity, relative gene expression and oxidative stress [169].

The neuroprotective effects of natural products against PD were studied using transgenic *C. elegans* PD models. An extract from the cultivated red seaweed *Chondrus crispus* decreased α-synuclein accumulation and reduced dopaminergic (DAergic) neurodegeneration induced by 6-hydroxydopamine hydrobromide (6-OHDA), an established neurodegeneration inducer. Moreover, slow movement and enhanced oxidative stress were recovered by supplementation with the extract via the upregulation of sod-3 and skn-1 [170]. Transgenic strains such as BZ555 (Pdat-1::GFP), UA57 (Pdat-1::GFP and Pdat-1::CAT-2), and NL5901 (unc-54p::α-synuclein::YFP) were utilized to further determine the protective effects of the methanol extracts of *Sorbus alnifolia* and *Holothuria scabra* extracts towards DA neurodegeneration [171,172]. Similar neuroprotection was observed with an ethanolic extract of rapeseed pomace, a rapeseed (canola) oil production byproduct, and *Decalepis hamiltonii* aqueous root extract with the induction of GST [173,174].

Regarding AD, various transgenic strains focusing on amyloid-β (Aβ) toxicity and tauopathies (reviewed extensively in [175,176,177]) are available for studying the effects of natural products. The ethanol extract of *Betula utilis* protects against Aβ toxicity via DAF-16 and SKN-1 [178]. The standard extract EGb 761 from *Ginkgo biloba* leaves inhibited Aβ aggregation via ROS attenuation [179]. Similar protection was achieved with the Chinese medicine *Salvia miltiorrhiza* water extract and the n-butanol extract of *Hedyotis diffusa* [180,181]. The protective effects of guarana (*Paullinia cupana*) hydroalcoholic extract in both AD and Huntington’s *C. elegans* disease models were dependent on SKN-1 and DAF-16 [160]. 

### 5.3. Digestive System

Distinct from higher eukaryotes, the digestive system of *C. elegans* is simplified, without a stomach, liver, pancreas and salivary gland. As a coelomic organism, the digestive system of *C. elegans* includes the buccal cavity, pharynx, pharyngeal-intestinal valve, intestine, rectum (or hindgut), anus and stomatointestinal muscle, of which the intestine is the major organ, accounting for one-third of the total somatic mass [182]. Regarding histology, the intestine comprises 20 epithelial cells to form long tubes through the body, and these cells are positioned as bilaterally symmetric pairs and form intestinal rings 1–9. Intestinal cells are all derived from a single progenitor cell E, the posterior daughter of EMS blastomeres. Then, Ea differentiates into rings int1-3 and int5, and Ep differentiates into rings int4 and int6-9 [183]. In the intestinal lumen of *C. elegans*, a prominent characteristic is the brush border formed by the membranous microvilli, and the glycocalyx located outside the microvilli is rich in glycoproteins providing a physical barrier to pathogens and allowing lumen contents to enter gut cells. During intestinal development, cell fates are primarily regulated by cell-autonomous transcriptional decisions. The transcription factor SNK-1 will upregulate the gene expression of *med*, which encodes GATA-type transcription factors. Among the seven GATA factors, MED-1, MED-2, END-1 and END-3 are only expressed before the 4E–8E cell phase, leaving three factors, ELT-2, ELT-4 and ELT7, expressed in the adult intestine for the regulation of development and function [184,185].

The intestine of *C. elegans* is involved in various physiological functions, such as digestion and metabolism, channels and transportation, defecation, endocytosis and exocytosis, stress response, host-pathogen interaction and vitellogenin regulation [186]. In *C. elegans*, lipofuscin accumulation is an interesting physiological phenomenon because it starts immediately after birth and continues throughout the lifespan [187]. Lipofuscin is a chemical waste material that originates from intracellular organelles and mainly comprises 30–70% proteins, 20–50% lipids, 4–7% carbohydrates, and small amounts of metals, such as iron. Lipofuscin deposits in the lysosomes of postmitotic cells in an irreversible manner and is associated with the pathological conditions of malnutrition, cancers and Batten’s disease, and others. Lipofuscin is shown as autofluorescence mainly in the intestine, under 330–380 nm ultraviolet excitation light and 420 nm barrier filter [188]. Natural products exhibit inhibitory effects on lipofuscin; for example, blueberry extract [189], epigallocatechin gallate from *Camellia sinensis* [190], broccoli-derived isothiocyanate sulforaphane [191], *Cleistocalyx nervosum var. paniala* fruit extracts [192] and naringin from the peel and fruit of *Citrus grandis*, *Citrus paradisi*, and *Citrus aurantium* [193] all reduced lipofuscin accumulation in the intestine of *C. elegans*. In contrast, the mycotoxin beauvericin was demonstrated to induce oxidative damage by increasing lipofuscin at a concentration of 10 μM [194].

To maintain the integrity of the intestinal barrier, *Genkwa Flos*, the flower of *Daphne genkwa* Sieb. et Zucc. affected the intestinal development of *C. elegans* by disrupting the apical junction, apical domain and microvilli of the intestine and prolonging the mean defecation cycle length, along with excessive ROS accumulation in the intestine [195]. 3,3′-Diindolylmethane, a metabolite of cruciferous vegetables, has been reported to have antioxidation capacity [196] and ameliorate *Pseudomonas aeruginosa*-induced intestinal inflammation and high permeability [197]. In the intestinal flora of *C. elegans*, a polysaccharide extracted from *Chlorella pyrenoidosa*, including seven monosaccharides, namely arabinose, mannose, rhamnose, galactose, glucose, galacturonic acid, and glucuronic acid, altered the enrichment of *Shewanella*, *Faecalibacterium*, *Vibrio*, and *Haemophilus*, and these changes were significantly correlated with oxidative damage to ROS, SOD and MDA [198]. For the innate immune system, water-soluble cranberry extract, which was beneficial to antioxidation, upregulated the expression of innate immune genes C23G10.1, *fmo-2*, *pqn-5*, *clec-46* and *clec-71*, thus resisting *Vibrio cholerae* infection [199].

### 5.4. Reproductive System

The most commonly reported indicator of fertility in *C. elegans* is the total number of progenies. For example, protocatechuic acid significantly reduces the total spawning number of *C. elegans* [200]. A reduced number of progenies was also found in *C. elegans* treated with mulberry leaf polyphenols [201]. This can be explained by the disposable soma theory of aging, which stresses that the limited cellular resources distribute reasonably between reproduction and the maintenance of somatic cells in the process of aging. Thus, lifespan extension and stress resistance are often associated with a reduction in fecundity [202,203]. However, many other compounds enhancing lifespan do not fit this hypothesis. For example, Jianpi-yangwei, a traditional Chinese medicine mainly composed of eight ingredients, *Panax ginseng* C.A.Mey. and *Radix Paeoniae Alba*, could increase the lifespan of *C. elegans* with a significant increase in the total progeny number [161]. In addition, numerous studies indicate that natural antioxidant products can extend the lifespan without reducing fertility in *C. elegans*, such as *Acanthopanax sessiliflorus* extract, naringin, and Gengnianchun [193,204,205]. Another reproductive indicator is the egg-laying time of *C. elegans*. Two plant polyphenols, gallic acid and ellagic acid could delay the beginning of egg deposition [206]. Protocatechuic acid also displayed delayed egg-laying [200]. Similarly, the self-fertile reproduction span of *C. elegans* treated with mulberry leaf polyphenols was significantly shorter than that of the control [201]. The deformation of reproductive organs is another indicator of reproduction toxicity. Several studies indicated that antioxidants protected the occurrence of genital malformation. Wu et al. [207] found that Hg exposure could lead to noticeable ROS accumulation in the gonads or vulva of nematodes, and the pretreatment with 100 g/mL of vitamin E at the L2 larval stage could effectively prevent the reduction in gonad arm length and the formation of abnormal vulvar structures.

### 5.5. Anti-Aging

Aging is a multifactorial process that is still poorly understood. Several theories have been proposed to explain the mechanisms of aging, and the most famous is Harman’s free radical theory of aging (FRTA) [58]. FRTA believes that free radicals cause the accumulation of oxidative damage in tissues and organs, leading to cell senescence. ROS are the most important and widely distributed oxygen-containing free radicals in cells. Excessive production of ROS may exceed the natural limit of the antioxidant capacity of cells. It will damage the DNA, membrane structure, protein and fat, further impairing cell function and increasing susceptibility to harmful external agents. Therefore, aging is partly the result of oxidative damage caused by ROS [208]. Studies have found that the increase in ROS induced by paraquat decreased the physiological longevity, chemotaxis and anti-heat stress ability of *C. elegans* [209]. Kim et al. [200] found that protocatechuic acid isolated from *Veronica peregrina* could attenuate intracellular ROS levels by upregulating antioxidant enzyme activities to enhance resistance and prolong lifespan.

On the one hand, oxidative damage in cells and tissues is inevitable with aging, so it is difficult to determine whether the damage is a cause or effect. Numerous studies using *C. elegans* to investigate the effect of life extension of natural products tend to find accompanying enhanced oxidation resistance. For example, the mean lifespan of *C. elegans* treated with the natural compound *Acanthopanax sessiliflorus* stem extract increased by 16.8% with increasing resistance to oxidative stress [205]. The relationship between antioxidation and antiaging remains unclear. In addition, the extension of longevity caused by natural products may not be the result of antioxidation and may simply instead be a concomitant effect. For example, overexpression of *sod-1*, which expresses SOD, increased the lifespan of *C. elegans*, not by removing ROS but by activating longevity-promoting transcription factors [210]. The classic traditional Chinese medicine Gengnianchun enhanced the lifespan of *C. elegans* via the insulin/IGF-1 signaling pathway, although antioxidant capacity increased at the same time [204]. Although high levels of free radicals are involved in cell damage and inflammation, they can also enhance cellular defenses through appropriate stress at low levels, known as mitohormesis. Bazopoulou et al. [211] proved that a transient increase in ROS during early development could improve stress resistance and extend lifespan in *C. elegans*, which challenges the rationality of FRTA. See Table 2 for some examples.

## 6. Conclusions and Future Perspectives

The oxidation and antioxidation of natural products have been assessed in cells, organs, tissues and animal models, and high-throughput screening methods based on machine learning. Each of them has obvious advantages and disadvantages. For example, during the nonclinical trial phase, we can predict the toxicity of a compound based on the quantitative structure-activity relationship and stimulate the static and dynamic interaction model between tens of thousands of protein pharmacophores and small molecules, including natural products, by means of web servers and softwares [214]. Benefitting from robust stimulation methods and high calculation efficiency, potential target protein candidates and aligned ligand poses were screened prior to experiments and clinical trials. However, relying solely on calculation methods will result in high false positive or false negative rates, and thus in vitro and in vivo experiments need to be carried out. The whole-animal models are high-cost and labor-intensive and occupy long experimental periods. Scientists have been looking for simpler animal models to achieve high-throughput screening. The Environmental Protection Agency (EPA) plan to gradually reduce the use of vertebrates, including rodents, rabbits and others, to evaluate the health effects of environmental pollution factors and chemicals [215]. New alternative methods are urgently required for modern toxicology and pharmacology research. As part of this effort, the Tox21 community, established by the U.S. National Toxicology Program, the EPA, and the National Institutes of Health, have used *C. elegans* to assess the toxicity of ToxCast Phase I and Phase II libraries containing 968 chemicals in total. The decreased larval growth and development are used as assessment parameters of chemical-induced toxicity in *C. elegans*. As a result, using *C. elegans* to predict rat or rabbit developmental toxicity resulted in high sensitivity and specificity [216]. In addition, previous studies have demonstrated the excellent predictive relationships between *C. elegans* and mammals in reproduction, locomotion, neurotoxicity and lethality [217,218,219]. After treating with natural products, the molecular targets for oxidative stress can be used, such as ROS, GSH level, the activity of endogenous antioxidants, enzymes, damaged biomacromolecule of lipid, protein and DNA. These abnormal pathological changes will further affect the physiological functions of immune, reproduction, aging, lifespan, organ development, signal transduction, and so on. An increasing number of assessments of the oxidative and antioxidative effects of natural products are being carried out in *C. elegans*, and appropriate evaluation indicators are being developed systematically.

As the first metazoan organism to complete genome sequencing, the cell differentiation lineage of *C. elegans* has been fully revealed, making it possible to modify the target genes and helping to facilitate molecular mechanism research [220]. According to the National Research Council, nearly 20,000 genes are reported in nematodes, with a high homology of 60–80% to humans [221]. Additionally, 12 out of the 17 known signaling transduction pathways in vertebrates are conserved in nematodes, namely Wnt/β-catenin, TGF-β receptor, small G-protein [Ras] linked, Notch-Delta, cytokine, apoptosis of cell death, receptor protein tyrosine phosphatase, large G-protein, integrin, cadherin, gap junction and ligand-gated cation channel pathways, which were involved in early and later development, as well as the physiological function of differentiated cells [222]. Large amounts of genetically modified worm strains provided convenient trails for genetic manipulation. As of 2019, the Gene Knockout Consortium had performed 15,000 open reading frames with putative null alleles by chemicals, radiations and transposons and thus obtained gene knockout and altered function strains for multidisciplinary research [223]. Series of oxidative stress-related transgenic nematodes are used for phenotype and mechanism studies. In *C. elegans*, the IIS and SKN-1/Nrf2 pathways mediate the transcription of downstream genes *sod-3*, *gst-10*, *gcs-1* and *sod-3*, etc., and then participate in phase II detoxification to maintain the balance between oxidation and antioxidation [224]. Thus far, the Caenorhabditis Genetics Center has owned plenty of transgenic nematodes of these two oxidative stress-related pathways. For example, the *daf-2* mutant strain DR1565 with the phenotypes of increased adult longevity and intrinsic thermotolerance can be used to investigate the role of *daf-2* in IIS pathway-involved oxidative stress regulation [225]. Moreover, the *daf-18*, *age-1*, *pdk-1*, *akt-1*, *akt-2*, *sgk-1*, *sir-2.1*, *jnk-1* and *daf-16* in this pathway have been knocked out, overexpressed or fluorescently labeled. To assess the natural products-triggered biological effects in *C. elegans*, the oxidation or antioxidation can be detected by lipofuscin accumulation, ROS production, oxidation or antioxidation enzymatic activity, etc. The toxicological or pharmacological endpoints can be set as survival (lethality, lifespan, healthspan), behavioral (pharyngeal pumping, locomotion, defecation, chemotaxis), growth and development (body length and width), reproduction (morphologic deformity, brood size, larval development) and the expression levels of genes and proteins [226].

Due to the advantages of simple culture and storage conditions, adequate cell differentiation profiling, a short experimental period and high genetic homology to humans, and others, *C. elegans* have been introduced into natural products research, with an increasing number of publications over the last two decades [227]. However, in investigating oxidation or antioxidation of natural products based on *C. elegans*, the natural products usually contain hundreds of compounds and induce a complicated process of absorption, distribution, metabolism and excretion (ADME), making it difficult to distinguish bioactive compounds. Many studies carried out in *C. elegans* have reported the beneficial or adverse oxidation and antioxidation effects of herbs, animals or microbes without further isolating single or several chemicals with bioactivity for verification, hence restricting the application and popularization of natural products. Another point of concern is that, as a free-living coelomic model organism, the organs of *C. elegans* are different than those of humans. For example, the main body structure of nematodes includes coelomocytes, cuticles, epithelial cells, muscles, nervous, and the alimentary, reproductive and excretory systems. Still, it lacks a liver, gallbladder, kidneys and blood system, which are very important for the ADME of natural products. For pharmacokinetic studies, in particular, the target organs of *C. elegans* are too small in size to isolate and detect the accumulation dosages, so the metabolic patterns are difficult to be assessed [228]. Based on the laboratory evaluation of the oxidation or antioxidation of natural products in *C. elegans*, there are still many concerning issues to be resolved to move towards real-world drug and toxicants supervision. *C. elegans* is an emerging model in the field of natural products and merits more attention for the assessment of biological effects, especially oxidation and antioxidation.

## Figures and Tables

**Table 1:** Advantages and disadvantages in the pharmacotoxicology of *C. elegans* and other models.

	Life Cycle	Metabolism	High-Throughput Screening	Costing	Live Imaging	Ethics and Welfare	3R	Phylogenetics	Cognitive Behavior	Homology with Human	Immune System	Genetic Manipulation
*C. elegans*	Very short lifespan (approximately 3 weeks), small body (1 mm), short reproductive cycle (3.5d) and large broodsize	As a multicellular organism composed of the brain, pharynx, intestine, gonads,	Available	Easy and low-cost in infrastructure and maintenance	Available	√	√	Different anatomical systems (no brain structure and immune system, etc.)	Extremely simple cognitive behaviors	Approximately 60–80% homologous genes to human; 12 of the 17 signal pathways in humans are conserved in nematodes	No immune system	Highly amenable to genetic manipulations
*Drosophila*	3 months	The metabolism of the whole body exists, lack of blood circulatory system, and blood–brain barrier, might cause inconsistent and unpredictable results when applied to humans	Available	Low-cost in infrastructure and maintenance	Unavailable	√	√	Simple and asymmetric brain structure	Relatively simple cognitive behaviors	Approximately 70% of the genes related to disease conditions in mammals are also present in Drosophila	Lack of an adaptive immune system	Highly amenable to genetic manipulations
Zebrafish	Fertilizing 200–300 eggs every 5–7 days, an equivalent longevity and generation time to mice (3–5 m)	Some major differences related to anatomy and physiology associated with an aquatic species, but most organs perform the same functions as their human counterparts and exhibit well-conserved physiology	Available	Relatively expensive in infrastructure and maintenance (compared to *Drosophila* and *C. elegans*)	Unavailable	√	√	A vertebrate animal model,	Limited cognitive behavioral assays	Approximately 70% homologous genes to human; over 80% of known human disease genes have orthologues in zebrafish	Complete immune system	Genetic tools yet to be comprehensive (compared to Drosophila and *C. elegans*)
Yeast	3 days	Unlikely as a suitable model	Available		Available	√	√	A single-celled organism	-	70% homologous genes to human; has no physiologic relevance to humans, but with many mitochondrial proteins that are orthologous to human proteins	-	Powerful genetic model,
Cell	Stable cell lines can be passed on for tens of generations	Cells alone are no longer metabolized in the whole body.	Available		Available	√	√	-	-	Human-derived cells as a research model	-	Amenable to genetic manipulations
Mammalian	Years	The metabolic process of the body is close to that of human beings.	Large-scale studies are limited	Costly in infrastructure and	Unavailable	×	×	Phylogenetically close to human	Complex cognitive analysis	Almost 100% human homolog genes found in rodents	Complete immune system	Costly in genetic manipulations

**Table 2:** Oxidation and antioxidation of natural products in the target organs of *C. elegans*.

	Target Organs	Natural Products	Stages of *C. elegans*	Time Intervals	Dosages	Culture Mediums	Assessment Indictors	References
Oxidation	Muscle	*Genkwa Flos* (GF), the flower of *Daphne genkwa* Sieb. et Zucc	L1 larvae of wild-type N2	From L1-larvae to young adult	0.12, 0.18 and 0.24 g/mL	NGM plates at 20 °C	Decreased head thrash and body bend	[195]
		Xanthotoxin	L1 larvae of wild-type N2	48 h	40, 60, 80, 100 and 120 mg/L	NGM plates at 25 °C	Decreased head thrash and body bend	[212]
	Nervous system	GF	L1 larvae of wild-type N2, *oxIS12*	From L1 to young adult	0.12–0.24 g/mL	NGM plates at 20 °C	Decreased locomotion behavior, deficits in AVL and DVB neurons, axonal degeneration and neuronal loss of D-type GABAergic motor neurons	[195]
	Digestive system	Mycotoxin beauvericin	L4 larvae of wild-type N2	Beauvericin treatment for 72 h followed by 24 h in compound-free medium	10, 50, 100 μM	Liquid NGM containing bovine serum albumin, streptomycin, and *E. coli*	Increased lipofuscin in intestine	[194]
		GF	L1 larvae of wild-type N2	From L1-larvae to young adult	0.24 g/mL	NGM plates at 20 °C	Disrupted the apical junction, apical domain and microvilli of intestine, prolonged mean defecation cycle length, increased ROS accumulation in intestine	[195]
		Xanthotoxin	L1 larvae of wild-type N2	48 h	40, 60, 80, 100 and 120 mg/L	NGM plates at 25 °C	Disordered and vacuolated cells in the intestinal cavity; shorter, denser, and disordered intestinal microvilli; longer intestinal epithelial cells; destroyed intestinal permeability; decreased number of intestinal bacteria	[212]
	Reproductive system	GF	L1 larvae of wild-type N2	From L1-larvae to young adult	0.18 g/mL and 0.24 g/mL	NGM plates at 20 °C	Decreased progeny number	[195]
		Xanthotoxin	L1 larvae of wild-type N2	From eggs to adults	40, 60, 80, 100 and 120 mg/L	NGM plates at 25 °C	Decreased hatchability, decreased progeny number, bulging and swelling vulva	[212]
		Mycotoxin beauvericin	L4 larvae of wild-type N2	7 days	100 μM	Liquid NGM containing bovine serum albumin, streptomycin, and *E. coli*	Decreased progeny number	[194]
Antioxidation	Muscle	Jianpi-yangwei	Synchronized wild-type N2	to 4th day of adulthood	150 μg/mL	NGM plates at 20 °C	Increased pharyngeal pumping rate	[161]
		Guarana extract	L1 larvae of wild-type N2	48 h	10 mg/mL and 50 mg/mL	NGM plates at 20 °C	Increased pharyngeal pumping rate	[159]
		*Gracilaria lemaneiformis* polysaccharide	Wild-type N2 at 1st day adulthood	5, 8, 10 days	250, 500, 1000 μg/mL	NGM plates at 20 °C	Increased pharyngeal pumping rate	[160]
		Protocatechuic acid	Synchronized wild-type N2	From L1-larvae to 4th day of adulthood	100 µM, 200 µM	NGM plates at 20 °C	Reduced pharyngeal pumping rate	[200]
	Nervous system	5-Desmethylnobiletin, a polymethoxyflavone	Adult age synchronized worms of wild-type N2	Until the assay	12.5–50 μM	NGM plates at 22 °C	Elevation in cholinergic transmission mediated through increased levels of ACh and activity of nicotinic acetylcholine receptors (nAChR).	[213]
		Whole body-ethyl acetate, body wall-ethyl acetate, and whole body-butanol fractions of Holothuria scabra extracts	BZ555 and NL5901 worms	L3 larve BZ555, 1 h. L1 larve NL9501, 72 h	500 μg/mL	NGM plates at 20 °C	Attenuated DA degeneration in BZ555, reduced α-synuclein aggregation in NL5901 worms induced by 6-OHDA	[171]
		*Lonicera japonica* extract or the combination composed of three major compounds (54 μg/mL chlorogenic acid, 15 μg/mL 1,5-dicaffeoylquinic acid and 7.5 μg/mL 1,3-dicaffeoylquinic acid)	CL4176 worms	treatment for 10 days	500 μg/mL	NGM plates 16 °C for 48 h, then shifted to 23 °C	Delayed paralysis	[73]
	Digestive system	Blueberry extract	Young adult stage of wild-type N2	5th day after treatment	50, 100, 200 mg/mL	NGM plates at 20 °C	Decreased lipofuscin in intestine	[189]
		Epigallocatechin gallate from *Camellia sinensis*	Hermaphrodites of the BA17 strain	Continuous 16 days on the day after hatching	220 μM	S-medium at 25 °C	Decreased lipofuscin in intestine	[190]
		Broccoli-derived isothiocyanate sulforaphane	L4 larvae	12th day after treatment	100 μM	NGM plates at 20 °C	Accelerated pharyngeal pump, decreased lipofuscin in intestine	[191]
		*Cleistocalyx nervosum var. paniala* Fruit Extracts	Wild-type N2	Continuous 5 days in age-synchronized young adult	20, 30 μg/mL	NGM plates at 15 °C	Decreased lipofuscin in intestine	[192]
		Naringin from the peel and fruit of *Citrus grandis*, *Citrus paradisi*, and *Citrus aurantium*	L4 larvae or young adults of wild-type N2	2nd and 5th days of adulthood	50 μM	NGM plates at 20 °C	Decreased lipofuscin in intestine	[193]
		3,3′-diindolylmethane, a metabolite of cruciferous vegetables	L4 larvae of wild-type N2	72 h	100 μM	NGM plates at 20 °C	Ameliorated the *Pseudomonas aeruginosa*-induced intestinal inflammation and high permeability	[197]
		Polysaccharides extracted from *Chlorella pyrenoidosa*	Synchronized wild-type N2	Continuous 5 days	10 mg/mL	NGM plates at 20 °C	Altered the enrichments of *Shewanella*, *Faecalibacterium*, *Vibrio*, and *Haemophilus*	[198]
		Water-soluble cranberry extract	L1 larvae of wild-type N2	Reached the young adult stage	Standardized for 2 mg/mL 4.0% proanthocyanidins	NGM plates at 25 °C	Upregulated the expression of innate immune genes C23G10.1, *fmo-2*, *pqn-5*, *clec-46* and *clec-71*	[199]
	Reproductive system	Jianpi-yangwei	Embryos of wild-type N2	Until they became adults	150 μg/mL	NGM plates at 20 °C	Increased progeny number	[161]
		Liangyi Gao	Embryos of wild-type N2	5 days	1 mg/mL	NGM plates at 20 °C	Increased progeny number	[163]
		*Gracilaria lemaneiformis* polysaccharide	L4 larvae of wild-type N2	5 days	1000 μg/mL	NGM plates at 20 °C	Increased reproduction duration	[160]
		Mulberry leaf polyphenols	Young adults	Until the last day of self-progeny production	25 mg/mL	NGM plates at 20 °C	Decreased progeny number	[201]
		Protocatechuic acid	L4 larvae	Until the last day of self-progeny production	100, 200 μM	NGM plates at 20 °C	Decreased progeny number; delayed spawning time	[200]
		Gallic acid and ellagic acid	L4 larvae of wild-type N2	85 h	300 μM gallic acid and 50 μM ellagic acid	NGM plates at 20 °C	Delayed the beginning of egg deposition	[206]

## Abbreviations

8-OHdG	8-hydroxy-2′-deoxyguanosine
AD	Alzheimer’s disease
ADME	absorption, distribution, metabolism, and excretion
AFB1	aflatoxin B1
CAT	catalase
*C. elegans*	*Caenorhabditis elegans*
*C. paliurus*	*Cyclocarya paliurus*
CRISPR	clustered regularly interspaced short palindromic repeats
DAF-16	forkhead box protein O
DSB	double strand break
ELISA	enzyme-linked immunosorbent assay
FoxO	Forkhead box O
FRTA	harman’s free radical theory of ageing
GC-MS	gas chromatography-mass spectrometry
GF	*Genkwa Flos*
GFP	green fluorescent protein
GLRX	glutaredoxin
GPX	glutathione peroxidase
GSH	glutathione
GST	glutathione S-transferase
H_2_DCF-DA	2′,7′-dichlorofluorescein diacetate
HNE	4-hydroxy-nonenal
H_2_O_2_	hydrogen peroxide
HO^•^	hydroxyl radical
HPLC	high-performance liquid chromatography
IGF-1	insulin/insulin-like growth factor-1
Keap1	Kelch-like ECH-associated protein 1
LOO^•^	peroxyl radicals
MAPK	p38 mitogen-activated protein kinase
MDA	malondialdehyde
MS-MS	tandem mass spectrometry
NER	nucleotide excision repair
Nrf2	nuclear factor erythroid 2-related factor 2
PD	Parkinson’s disease
PI3K	phosphatidylinositol-3-OH kinase
PRDX	peroxiredoxins
PUFA	polyunsaturated fatty acid
RNAi	RNA interference
ROS	reactive oxygen species
SKN-1	skinhead-1
SOD	superoxide dismutase
TOR	target-of-rapamycin
TRX	thioredoxins
